# E-cadherin Downregulation and microRNAs in Sporadic Intestinal-Type Gastric Cancer

**DOI:** 10.3390/ijms20184452

**Published:** 2019-09-10

**Authors:** Tania Rossi, Gianluca Tedaldi, Elisabetta Petracci, Raefa Abou Khouzam, Guglielmina Nadia Ranzani, Paolo Morgagni, Luca Saragoni, Manlio Monti, Daniele Calistri, Paola Ulivi, Chiara Molinari

**Affiliations:** 1 Biosciences Laboratory, Istituto Scientifico Romagnolo per lo Studio e la Cura dei Tumori (IRST) IRCCS, 47014 Meldola, Italy; 2 Unit of Biostatistics and Clinical Trials, Istituto Scientifico Romagnolo per lo Studio e la Cura dei Tumori (IRST) IRCCS, 47014 Meldola, Italy; 3 Department of Biology and Biotechnology, University of Pavia, 27100 Pavia, Italy; 4 General and Oncologic Surgery, Department of Surgery, G.B. Morgagni L.Pierantoni General Hospital, AUSL Romagna, 47121 Forlì, Italy; 5 Department of Pathology, AUSL Romagna, Morgagni-Pierantoni Hospital, 47121 Forlì, Italy; 6 Department of Medical Oncology, Istituto Scientifico Romagnolo per lo Studio e la Cura dei Tumori (IRST) IRCCS, 47014 Meldola, Italy

**Keywords:** E-cadherin, *CDH1*, intestinal-type gastric cancer, microRNAs

## Abstract

*CDH1* gene, encoding E-cadherin, is a tumor suppressor gene frequently altered in gastric cancers (GCs) of both diffuse (DGC) and intestinal (IGC) histotypes, albeit through different mechanisms. The study aimed to characterize *CDH1* expression in sporadic IGC and to investigate whether microRNAs (miRs) are involved in its transcriptional control. We evaluated *CDH1* expression by quantitative real-time PCR (RT-qPCR) in 33 IGC patients and found a significant downregulation in tumor tissues compared to normal counterparts (*p*-value = 0.025). Moreover, 14 miRs, predicted to be involved in *CDH1* regulation in both a direct and indirect manner, were selected and analyzed by RT-qPCR in an independent case series of 17 IGCs and matched normal tissues. miR-101, miR-26b, and miR-200c emerged as significantly downregulated and were confirmed in the case series of 33 patients (*p*-value < 0.001). Finally, we evaluated *EZH2* expression, a target of both miR-101 and miR-26b, which showed significant upregulation in IGCs (*p*-value = 0.005). A significant inverse correlation was observed between *EZH2* overexpression and *CDH1*, miR-101, and miR-26b levels (*p*-value < 0.001). Our results reinforce the link between *CDH1* and IGC, highlighting the role of miRs in its transcriptional control and improving our understanding of GC subtypes and biomarkers.

## 1. Introduction

Gastric cancer (GC) is the fifth most common cancer and the third cause of cancer-related death worldwide [1]. Lauren’s classification, firstly reported in 1965, is currently used to distinguish two gastric adenocarcinoma subtypes based on histological and clinical features: intestinal-type gastric cancer (IGC) and diffuse-type gastric cancer (DGC) [2]. It is widely accepted that IGC and DGC represent distinct disease entities with different epidemiology, etiology, carcinogenesis, progression and, to some extent, biological behaviors [3].

*CDH1* gene encodes E-cadherin (Epithelial-cadherin), a Ca^2+^-dependent transmembrane glycoprotein involved in cell−cell adhesion maintenance in epithelia [4,5]. Accordingly, E-cadherin plays a crucial role in epithelium−mesenchymal transition (EMT): its underexpression reduces cell-cell cohesion, making it possible for tumor cells to dissociate from primary tissue, invade surrounding tissues and disseminate to other sites [6,7,8]. Indeed, numerous studies have highlighted E-cadherin as a critical tumor suppressor in several carcinomas, including GC [9,10].

*CDH1* is especially altered in hereditary diffuse GC (HDGC), where complete loss of protein expression often occurs due to a germline lesion and to a second hit, following Knudson’s theory of tumor suppressor gene inactivation [11]. With regard to sporadic tumors, which account for 90% of GCs, it has been reported that epigenetic and structural alterations are as frequent in IGC as in DGC, suggesting histotype independence [12]. Nonetheless, *CDH1* status in IGC is not as extensively studied as in DGC.

In the last decade, the focus has been placed on alternative mechanisms capable of modifying E-cadherin expression, including epigenetic control exerted by noncoding transcripts. MicroRNAs (miRs) are small non-coding RNAs, which negatively regulate gene expression and orchestrate pathways involved in cell-cycle control, proliferation, apoptosis, angiogenesis, metastasis, and DNA-damage response in cancers, including GC [13,14,15,16,17,18,19]. Of note, by downregulating the expression of target cancer-related genes, several miRs have been shown to be directly involved in carcinogenic processes, including EMT [20,21]. In this scenario, *CDH1* transcription is often susceptible to expression control of miRs and long noncoding RNAs [22]. This occurs in both direct and indirect manners due to the number of pathways regulating *CDH1* expression through the activity of Snail, Slug, ZEB1/2, EZH2, and Twist transcription factors [23,24]. 

In this study, we aimed at analyzing the regulation of *CDH1* expression in a case series of IGC and matched normal tissue samples, by focusing on the role of miRs that target *CDH1* gene, either directly or indirectly.

## 2. Results

### 2.1. CDH1 is Significantly Downregulated in IGC

In the present study, a cohort of 33 patients affected by IGC was retrospectively enrolled. Clinicopathological data are summarized in Table 1. 

**Figure 1:**
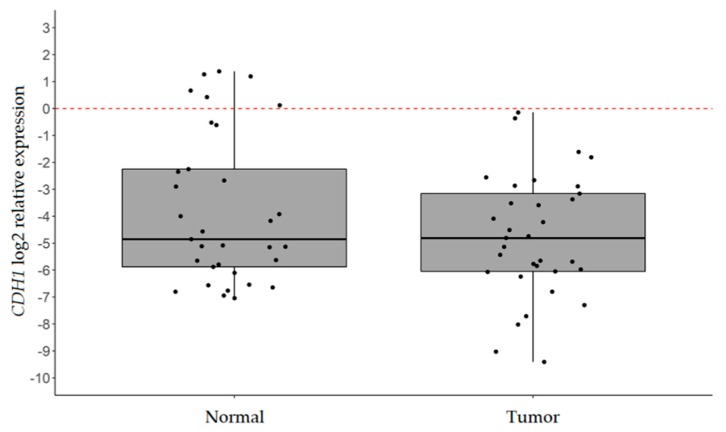
*CDH1* expression analysis by real-time (RT)-qPCR. Box plots of the log2 relative expression (2^−ΔCt^) of *CDH1* in neoplastic and matched normal tissues of 33 IGC patients. *B2M* was used as the internal control. *p*-value = 0.025 (paired student’s *t*-test).

*CDH1* transcript levels were analyzed in fresh-frozen neoplastic tissues and normal counterparts by reverse transcription quantitative real-time PCR (RT-qPCR). As shown in Figure 1, we observed a statistically significant decrease in *CDH1* mRNA in tumor samples with respect to normal adjacent tissues (log2 mean relative expression −4.76 ± 2.27 vs −3.77 ± 2.79; *p*-value = 0.025). In particular, *CDH1* downregulation was observed in 14 out of 33 samples (42.4%; fold change (FC) ≤ 0.50; Table S2). No significant association between patients’ clinical pathological features and *CDH1* expression was observed.

### 2.2. Explorative Analysis Suggests that the Expression of miR-101, miR-26b, and miR-200c is Altered in IGC

In order to investigate the miR-related transcriptional control of E-cadherin in IGC, we used a combined in silico and literature-based approach to identify miRs known to downregulate *CDH1*, either directly or indirectly. This approach gave rise to a list of 14 miRs (Table 2). A complete description of miR selection criteria is summarized in the Supplementary Materials.

Due to the limited amount of RNA from 33 IGC patients, an explorative analysis of the expression profiles of the 14 selected miRs was performed on tumor and normal samples from an independent case series of 17 IGCs, in order to identify the miRs that were most differentially expressed in IGC. Eight out of the 14 miRs could be successfully quantified (miR-141, miR-429, miR-200a, miR-200b, miR-200c, miR-101, miR-26b, and miR-23a). In particular, miR-101 (*p*-value = 0.0023) and miR-26b (*p*-value = 0.0016) expression levels were significantly lower in tumors than in normal tissues, while miR-200c showed borderline significance (*p*-value = 0.0537).

### 2.3. miR-101, miR-26b, and miR-200c are Confirmed to be Significantly Downregulated in IGC

The levels of the 3 miRs differentially expressed in the exploratory case series were analyzed in the case series of 33 IGC patients by RT-qPCR using specific TaqMan gene expression assays.

**Figure 2:**
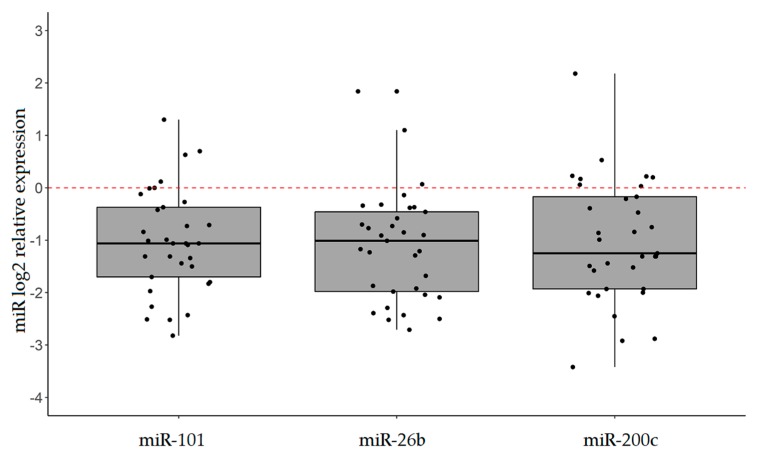
miR expression analysis by RT-qPCR. Box plots of miR-101, miR-26b, and miR-200c expression (log2 FC) in 33 intestinal gastric cancer (IGC) patients. RNU6 was used as the internal control. *p*-value < 0.001 for all miRs (paired student *t*-test).

As shown in Figure 2, miR-101, miR-26b, and miR-200c proved to be significantly less expressed in tumor tissues with respect to normal counterparts (mean FC = 0.50; *p*-value < 0.001).

**Figure 3:**
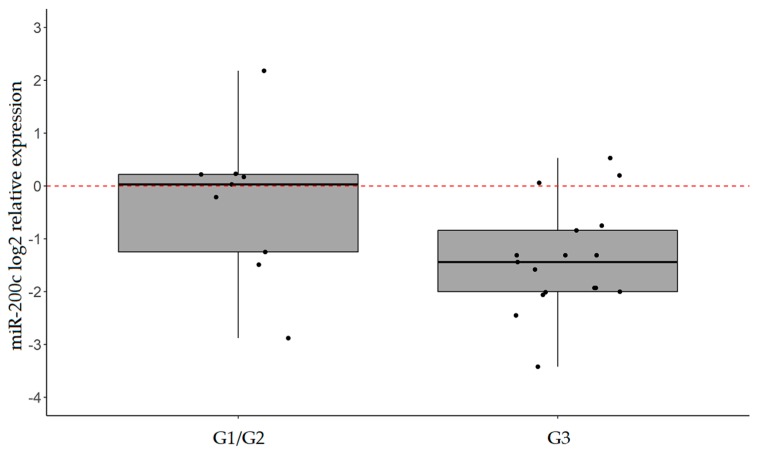
miR-200c and tumor grade. Box plots of miR-200c expression (log2 FC) in tumors grouped according to grade. *p*-value = 0.049 (Mann−Whitney test).

In particular, miR-101, miR-26b, and miR-200c were found to be downregulated in 57.6%, 51.5%, and 51.5% of IGCs, respectively (FC ≤ 0.50). Of note, miR-200c levels were significantly associated with tumor grade, being lower in G3 tumors compared to G1/G2 disease (*p*-value = 0.049; Figure 3). No significant association was observed between miR-101 or miR-26b expression and clinical pathological characteristics of patients.

### 2.4. EZH2 Expression Levels are Increased in IGC Specimens Compared with the Normal Counterpart and Inversely Associated with CDH1 Expression

**Figure 4:**
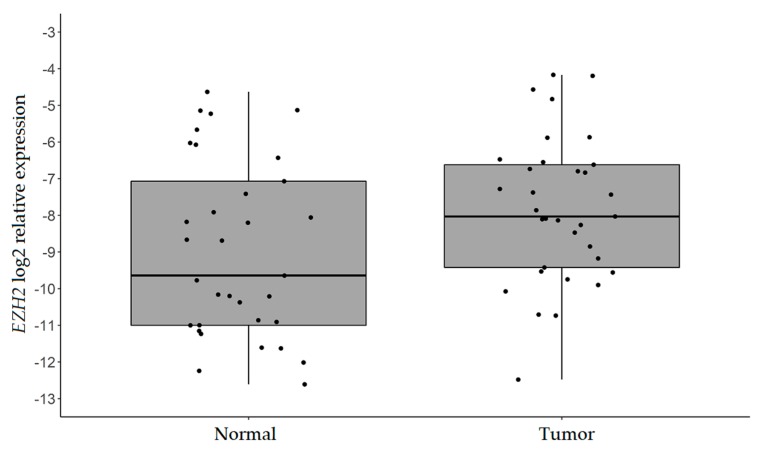
*EZH2* expression analysis by RT-qPCR. Box plots of the log2 relative expression (2^−ΔCt^) of *EZH2* in neoplastic and matched normal tissues of 33 IGC patients. *B2M* was used as the internal control. *p*-value = 0.005 (paired student’s *t*-test).

Given that *EZH2* is a target of both miR-101 and miR-26b and an important *CDH1* inhibitor, we evaluated *EZH2* transcript levels to verify if its upregulation could contribute to *CDH1* downregulation in our case series. Despite being expressed at very low levels in both tumor and normal samples, *EZH2* was significantly more expressed in IGC tissues compared to normal tissues (log2 mean relative expression −7.84 ± 1.99 vs. −8.94 ± 2.41; *p*-value = 0.005; Figure 4).

**Figure 5:**
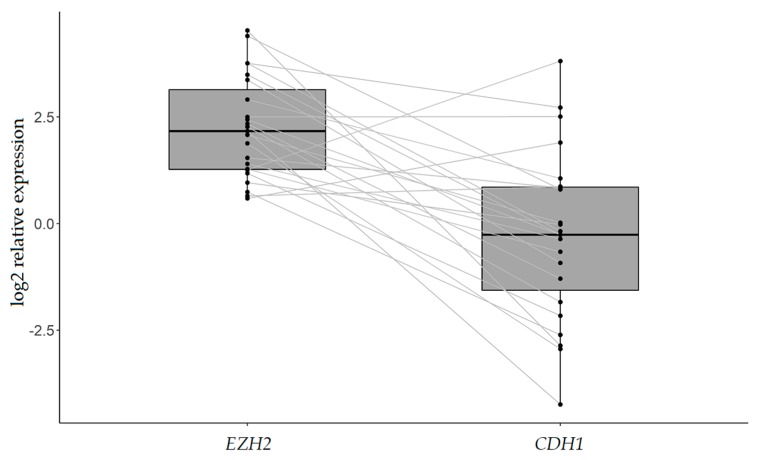
*EZH2* upregulation and its association with *CDH1* expression. Box plots of *CDH1* expression in relation to *EZH2* expression in 23 IGC patients with *EZH2* upregulation. Relative expression is measured as log2 FC. *p*-value *<* 0.001 (Mann−Whitney test).

**Figure 6:**
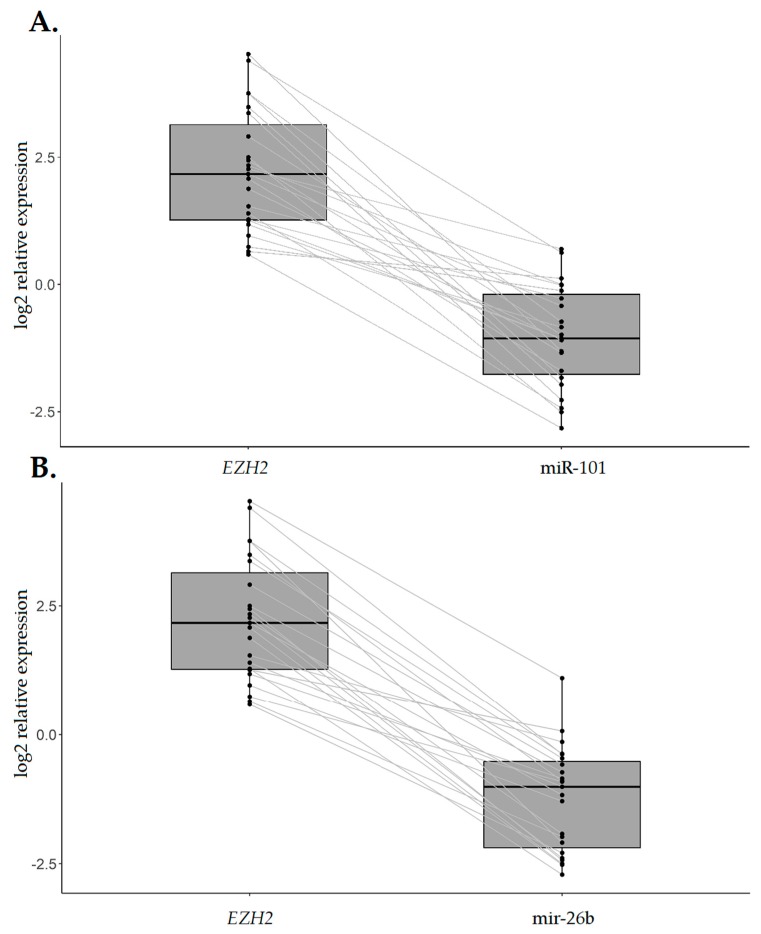
*EZH2* overexpression and its association with miR-101 and miR-26b expression. Box plots of miR-101 (**A**) and miR-26b (**B**) expression in relation to *EZH2* expression in 23 IGC patients with *EZH2* upregulation. Relative expression is measured as log2 FC. *p*-value < 0.001 (Mann−Whitney test).

In tumors with *EZH2* overexpression (*n* = 23), a significant association with *CDH1* decrease was observed (*p*-value < 0.001) (Figure 5), whereas the same was not observed for the remaining tumors with *EZH2* downregulation or normal expression (*p*-value = 0.160). Similarly, a statistically significant inverse association was found between *EZH2* upregulation and miR-101/miR-26b expression (Figure 6A,B).

## 3. Discussion

E-cadherin is a transmembrane glycoprotein that plays a pivotal role in maintaining epithelial architecture and cell polarity [5]. In the last few decades, E-cadherin tumor suppressor function has emerged in different epithelial tumors, including GC, and its downregulation has been observed during neoplastic progression and in association with tumor invasion [25,26,27,28] and metastasis [8,29]. In 1998, Karayiannakis et al. described aberrant or absent E-cadherin protein expression in both IGC and DGC [30]. It has been reported that *CDH1* alterations, both structural and epigenetic, occur in almost one-third of sporadic GCs, with slightly higher frequencies in DGC than in IGC [12]. More recently, data mining of different repositories indicates that *CDH1* is the second gene related to IGC [31], raising the question of the mechanisms that contribute to its expression in this histotype. In recent years, miRs have emerged as promoters and suppressors of carcinogenesis and metastasis in many types of cancers [32]. With regard to GC, a wide range of miRs have been associated with *Helicobacter pylori* (HP)-related infection, a well-established event in IGC carcinogenesis [33]. Interestingly, E-cadherin downregulation has been described in concomitance with HP infection-derived neutrophil infiltration [34]. Moreover, it has been shown that several miRs are involved in epithelial-mesenchymal transition (EMT), modulating E-cadherin expression by directly targeting *CDH1* or acting on one or more of its transcription factors, including EZH2, ZEB1, ZEB2, and Slug [21]. Interestingly, these pathways emerged to have a role also in chemotherapeutic resistance in GC, *CDH1* direct or indirect restoration may be a useful way to reduce it in such tumors [35].

In this study, we aimed to characterize the *CDH1* expression levels and its transcriptional regulation by investigating the impact of miRs on IGC carcinogenesis. We found that *CDH1* is transcriptionally downregulated in 42.4% of IGCs, further confirming the importance of *CDH1* downregulation in this gastric cancer histotype [36].

Among the miRs filtered by in silico analysis as direct/indirect regulators of *CDH1* expression, miR-34c, miR-506, miR-217, miR-199a, miR-153, and miR-544 were undetectable in both normal and tumor samples from a cohort of 17 IGCs. Conversely, among evaluable miRs, miR-101, miR-26b, and miR-200c proved to be significantly downregulated in IGC compared to control tissue both in this exploratory cohort and in our 33-patient case series.

miR-101 has been reported to act as a tumor suppressor by targeting *CDH1* inhibitors, such as *ZEB1/ZEB2* and *EZH2* in different tumors [37,38], including GC [39,40]. Low levels of miR-101 in plasma have been reported to be associated with GC progression [41] and HP-induced inflammation [42,43]. Our data, in accordance with previous results from our group [36], showed that miR-101 is significantly downregulated in IGC patients. However, likely due to the limited number of patients, no association between miR-101 expression and clinical pathological parameters, including HP infection, emerged from this study.

miR-26b is expressed at low levels in GC, and its downregulation is associated with a higher TNM classification and shorter survival [44]. Several studies have shown that miR-26b, like miR-101, inhibits *EZH2* expression leading to *CDH1* downregulation in many tissues, including GC [45]. In agreement with these findings, we observed a significant downregulation of miR-26b in tumor specimens compared to the normal counterparts.

miR-200 family members are known as transcriptional repressors of E-cadherin through the regulation of *ZEB1* and *ZEB2*. In gastric cell lines, miR-200 family members are markedly downregulated during EMT with a concomitant decrease in E-cadherin, and their lower expression has been associated with poor prognosis in patients [46,47,48]. There is evidence that miR-200 family members act as tumor suppressors in GC [49,50]. Among these, miR-200c showed a significantly lower expression in neoplastic tissue than normal gastric mucosa in our study. Moreover, in line with previous findings [51], its expression was significantly associated with tumor grade, being lower in poorly differentiated GC (G3) than in more differentiated tumors (G1 and G2). These observations reveal that miR-200c is potentially involved in IGC cell differentiation status.

Overall, although miR-101, miR-26b, and miR-200c were downregulated in more than half of our patients, only half of them showed concomitant *CDH1* downregulation, indicating a more complex role in regulatory networks for these miRs.

We also found higher *EZH2* expression levels in IGC specimens and we detected a statistically significant *CDH1* downregulation in IGC patients showing *EZH2* upregulation. In accordance with previous studies suggesting that miRs targeting *EZH2* may be associated with the perturbation of E-cadherin expression [39], we observed a statistically significant inverse association between *EZH2* and miR-101/26b expression levels.

In conclusion, our results reinforced the emerging link between E-cadherin and intestinal-type GC and confirmed the role of *EZH2* as a regulator of *CDH1* expression. Furthermore, our findings highlighted the potential of some specific miRs to be exploited as molecular markers of tumorigenesis and aggressiveness in this specific cancer histotype.

However, the small number of patients involved was not sufficient for us to identify any significant correlation between the analyzed markers, making it necessary to perform these studies on larger cohorts in order to further refine the biomarkers selection and to identify new therapeutic targets in IGC.

## 4. Materials and Methods

### 4.1. Samples

Thirty-three patients with GC submitted to surgical resection between 2007 and 2017 and classified as IGC by an expert pathologist, according to Lauren’s classification, were included in this study. Patients treated with neoadjuvant therapy were not considered. Surgical samples were immediately cryopreserved after resection. Fresh-frozen tumor tissue and matched normal gastric epithelium samples were stored by the Biological Resources Center (CRB) of Meldola (Italy) until the molecular analyses were performed. All subjects gave written informed consent to the conservation and use of the samples for research purposes. The study was conducted in accordance with the Declaration of Helsinki and the protocol was approved by the Romagna Ethics Committee (CEROM) of Meldola (IRSTB062, approved on 21 February 2018).

### 4.2. RNA Extraction

Total RNA extraction was performed on tumor and normal samples using TRIzol (Invitrogen, Carlsbad, CA, USA) in accordance with manufacturer’s instructions. Four micrograms of extracted RNA were treated with DNase and purified by using RNeasy MinElute Cleanup kit (Qiagen, Hilden, Germany). All the steps indicated in the protocol were followed, however, to allow recovery of miRs, 950 µL of ethanol 96−100% were used before column filtration. RNA was quantified by Spectrophotometer Nanodrop-ND-1000 (Thermo Fisher Scientific, Waltham, MA, USA).

### 4.3. Gene Expression Analyses

One microgram of purified RNA was reverse transcribed using an iScript cDNA synthesis kit (Bio-Rad Laboratories, Hercules, CA, USA) according to the manufacturer’s instructions. The reactions were run with the following thermal conditions: 25 °C for 5 min, 42 °C for 30 min, and 85 °C for 5 min. RT-qPCR reactions were carried out to assess the expression levels of *CDH1* (Life Technologies, Carlsbad, CA, USA) and *EZH2* (Integrated DNA Technologies, Coralville, IA, USA). β2 microglobulin (*B2M*, Life Technologies) was used as the endogenous control for normalization. The reactions were run in duplicate on 40 ng of cDNA on ABI 7500 RT-qPCR system (Applied Biosystems, Foster City, CA, USA) under the following thermal conditions: 95 °C for 10 min; 40 cycles of 95 °C for 15 s and 60 °C for 60 s. Expression levels of the target genes were obtained by normalizing the results using the endogenous control *B2M*. Relative expression was quantified using the comparative 2^−ΔCt^ method and FC values in gene expression were calculated using the 2^−ΔΔCt^ method [52,53]. An FC ≤0.50 and ≥1.5 were used as cut off for downregulation and upregulation, respectively.

### 4.4. miR Analyses

Six hundred nanograms of purified RNA from tumor tissue and the normal counterpart were converted into cDNA by TaqMan microRNA RT kit (Applied Biosystems) following the manufacturer’s protocol. The reverse transcription reaction was performed using a custom-made primer pool of miRs of interest and the following thermal protocol: 16 °C for 30 min, 42 °C for 30 min, and 85 °C for 5 min. miRs expression was assessed using custom TaqMan microRNA 96-well plates (Life Technologies) with lyophilized assays of selected miRs. A single 96-well plate was sufficient for miR profiling of one cancer sample and the normal counterpart in triplicate and *RNU6* was used as the endogenous reference. RT-qPCR reactions were run on a ABI 7500 real-time PCR System (Applied Biosystems) applying the following thermal protocol: 95 °C for 10 min, 40 cycles of 95 °C for 15 s, and 60 °C for 60 s. Relative expression and FC were calculated as for mRNA.

RT-qPCR was used to confirm the differentially expressed miRs by single assays and the same experimental conditions as reported previously.

### 4.5. Statistical Analyses

Data were summarized using mean ± standard deviation for continuous variables and absolute frequency and percentage for categorical variables. A paired student t-test was used to compare expression levels between tumor and normal samples, while the Mann−Whitney tests were used to compare expression levels between patient groups defined by clinical characteristics (*p*-values < 0.05 were considered statistically significant).

## Figures and Tables

**Table 1:** Clinical pathological characteristics of patients. Tumor staging was done based on the tumor (T), lymph node (N), and metastasis (M) system. N/A: not available.

Parameter	Total	
	*n* = 33	%
Sex		
F	13	39.4
M	20	60.6
Age, years		
<77	15	45.5
≥77	18	54.5
T		
1	2	6.1
2	12	36.4
3	11	33.3
4	6	18.2
N/A	2	6.1
N		
0	13	39.4
+	18	54.5
N/A	2	6.1
M		
0	11	33.3
1	2	6.1
X	7	21.2
N/A	13	39.4
Grade		
1	1	3.0
2	8	24.2
3	17	51.5
N/A	7	21.2
Tumor site		
Cardia	3	9.1
Fundus	1	3.0
Body	8	24.2
Antrum	10	30.3
N/A	11	33.3
Tumor size (cm)		
<5	16	48.9
≥5	17	51.1
*Helicobacter pylori*		
Positive	16	48.5
Negative	14	42.4
N/A	3	9.1

**Table 2:** Selected miRs, target genes, and rationale of selection.

miR	In Silico Prediction				Experimental Evidence
	Target Gene	miRTarBase	miRDB	TargetScan	miR-Target Interaction (+) or miR Expression Deregulation [ref.] in GC
miR-506	*SNAI2*	+	+	+	+
miR-141	*ZEB1*	+	+	+	+
	*ZEB2*	+	+	+	+
miR-217	*EZH2*	+	+	+	+
miR-429	*EZH2*	+	+	+	+
miR-199a	*CDH1*	+	+	+	+
miR-200a	*ZEB1*	+	+	+	+
	*ZEB2*	+	+	+	+
miR-200b	*ZEB1*	+	+	+	+
	*ZEB2*	+	+	+	+
miR-200c	*ZEB1*	+	+	+	+
	*ZEB2*	+	+	+	+
	*SUZ12*	+	+	+	+
miR-101	*EZH2*	+	+	+	+
	*ZEB1*	+	+	+	+
miR-153	*SNAI1*	+	+	+	+
miR-26b	*EZH2*	+	+	+	[15,16]
miR-23a	*CDH1*	+	+	+	+
miR-544	*CDH1*	+	+	+	+
miR-34c	*SNAI1*	+	+	+	[17]

## Supplementary Materials

Supplementary materials can be found at https://www.mdpi.com/1422-0067/20/18/4452/s1.
