# Supplementary material for: E-cadherin Downregulation and microRNAs in Sporadic Intestinal-Type Gastric Cancer
Source: Int J Mol Sci. 2019 Sep 10;20(18):4452. doi: 10.3390/ijms20184452 (PMC6769612; doi:10.3390/ijms20184452)
Supplement: Supplementary file 1 [file ijms-20-04452-s001.pdf]

Supplementary materials

Selection of *CDH1*-regulating miRNAs in gastric cancer

An *in-silico* analysis was executed to determine miRNAs that can potentially target the 3'UTR of *CDH1* and its regulators *SNAI1, SNAI2, TWIST1, ZEBI, ZEB2, EZH2* and *SUZ12*. To that purpose, the following three databases and predictive bioinformatics tools were utilized:

1. miRTarBase v6.0 (http://miRTarBase.mbc.nctu.edu.tw/), which is the most comprehensively annotated online database reporting experimentally validated microRNA-target interactions (MTIs). The database has thus far accumulated 4,966 articles, 7,439 strongly validated MTIs (through reporter assays or western blots) and 348,007 MTIs from crosslinking and immunoprecipitation sequencing [1]. In addition to that, in version 6 of the database you can query miR-target in disease and get as a result a list of experimentally tested miRNAs and their targets that have been reported in that specific disease.
2. TargetScan v7.1 (http://www.targetscan.org/) that is an online site in which biological targets of miRNAs are predicted based on the presence of conserved 8mer, 7mer and 6mer sites matching the seed region of each miRNA [2]. It provides accurate rankings of the predicted targets for miRNAs based on either the probability of evolutionarily conserved targeting (P_{CT} scores) [3] or the predicted efficacy of targeting (context+++ scores) [4]. TargetScanHuman is based on matches to human 3' UTRs and their orthologs, as indicated by UCSC (University of California, Santa Cruz) whole-genome alignments.
3. miRDB v5 (http://mirdb.org/miRDB/), which is an online database for miRNA target prediction and functional annotations. Predictions on MTIs can be queried by miRNA names or by gene-target information. The targets reported in this database have been derived from the bioinformatics tool MirTarget v3. This tool was developed upon the analysis of thousands of MTIs from high-throughput sequencing experiments, which allowed the identification of common features associated with miRNA target binding. These features were then acquired by machine learning methods to predict miRNA targets [5,6].

This extensive analysis was combined with a search of the literature regarding miRNAs' expression in gastric cancer to guide the selection of a list of miRNAs to be differentially quantified in paired normal and tumor tissue of the IGC patients.

Table S1: List of the miRs selected from literature on the basis of their involvement in direct or indirect E-cadherin repression.

miRbase Id	Assay ID/Name	Mature miR sequence
hsa-miR-506	001050	UAAGGCACCCUUCUGAGUAGA
hsa-miR-141-3p	000463	UAACACUGUCUGGUAAAGAUGG
hsa-miR-217	002337	UACUGCAUCAGGAACUGAUUGGA
hsa-miR-429	001024	UAAUACUGUCUGGUAAAACCGU
hsa-miR-199a-5p	000498	CCCAGUGUUCAGACUACCUGUUC
hsa-miR-200a-3p	000502	UAACACUGUCUGGUAACGAUGU
hsa-miR-200b-3p	002251	UAAUACUGCCUGGUAAUGAUGA
hsa-miR-200c-3p	002300	UAAUACUGCCGGGUAAUGAUGGA
hsa-miR-101-3p	002253	UACAGUACUGUGAUAACUGAA
hsa-miR-153-3p	001191	UUGCAUAGUCACAAAAGUGAUC
hsa-miR-26b-5p	000407	UUCAAGUAAUUCAGGAUAGGU
hsa-miR-23a-3p	000399	AUCACAUUGCCAGGGAUUUCC
hsa-miR-544a	002265	AUUCUGCAUUUUUAGCAAGUUC
hsa-miR-34c-5p	000428	AGGCAGUGUAGUUAGCUGAUUGC
U6 snRNA	001973	GTGCTCGCTTCGGCAGCACATATACTAAAATTGGAACGA TACAGAGAAGATTAGCATGGCCCCTGCGCAAGGATGACA CGCAAATTCGTGAAGCGTTCCATATTTT

Table S2. Expression levels of target mRNA and miR in the case series of 33 patients. The cases are ordered based on *CDH1* FC (Fold Change). N=Normal; T=Tumor.

Patient ID	FC *CDH1*	2-ΔCT *CDH1*	FC *EZH2*	2-ΔCT *EZH2*	FC miR-101	2-ΔCT miR-101	FC miR-26b	2-ΔCT miR-26b	FC miR-200c	2-ΔCT miR-200c
05/09N		1,33793		0,01160		0,01105		0,17678		0,10366
05/09T	**0,0280**	0,03742	0,5548	0,00643	0,4796	0,00530	0,4323	0,07642	1,1487	0,11908
16/09N		0,06250		0,00247		0,01184		0,69255		0,46009
16/09K	**0,0306**	0,00191	0,2415	0,00060	0,1743	0,00206	0,2736	0,18946	0,8888	0,40895
18/11N		2,29740		0,01978		0,02256		0,14865		0,26794
18/11T	**0,0421**	0,09672	0,2932	0,00580	0,3686	0,00832	0,2432	0,03615	0,4204	0,11266
12/08N		2,60268		0,00744		0,05831		0,76844		1,14076
12/08T	**0,0529**	0,13774	5,6569	0,04210	0,1756	0,01024	0,1856	0,14260	0,2624	0,29937
17/11N		1,59107		0,02664		0,01296		0,20166		0,25000
17/11T	**0,0548**	0,08717	0,3816	0,01017	0,7738	0,01003	0,6156	0,12414	0,8645	0,21613
06/07N		2,41162		0,02836		0,00572		0,11344		0,12674
06/07T	**0,0559**	0,13490	0,1518	0,00430	0,2872	0,00164	0,3121	0,03540	0,2398	0,03040
14/09N		0,65520		0,01541		0,00375		0,15177		0,26794
14/09K	**0,0819**	0,05366	0,6926	0,01067	0,6113	0,00229	0,7900	0,11991	0,7220	0,19345
02/14N		0,02956		0,00024		0,00635		0,11908		0,17556
02/14K	**0,1303**	0,00385	2,4284	0,00059	0,9931	0,00630	0,7270	0,08657	0,0934	0,01640
10/16N		0,03467		0,00021		0,00416		0,05831		0,09151
10/16K	**0,1377**	0,00478	5,0630	0,00105	0,3078	0,00128	0,2643	0,01541	0,2500	0,02288
09/15N		0,00897		0,00032		0,00274		0,04904		0,06792
09/15K	**0,1638**	0,00147	4,2281	0,00135	0,4796	0,00132	0,5359	0,02628	0,3686	0,02503
04/12N		0,19615		0,00416		0,00596		0,23651		0,38422
04/12K	**0,2238**	0,04389	2,2658	0,00942	0,4796	0,00286	0,5322	0,12587	0,4033	0,15496
12/12N		0,06606		0,00242		0,00436		0,17924		0,20027
12/12K	**0,2253**	0,01488	1,3472	0,00326	0,4033	0,00176	0,4263	0,07642	1,1251	0,22531
09/07N		0,21022		0,00375		0,01360		0,19345		0,21022
09/07T	**0,2793**	0,05872	2,3950	0,00897	0,6029	0,00820	0,6029	0,11663	1,1728	0,24656
06/09N		0,69737		0,00340		0,01640		0,22531		0,48633
06/09T	**0,4090**	0,28519	10,3388	0,03516	0,5035	0,00826	0,9075	0,20448	0,4033	0,19615
19/17N		0,02856		0,00115		0,00515		0,12500		0,19078
19/17K	0,5285	0,01509	1,5052	0,00172	1,5476	0,00798	0,7738	0,09672	0,4033	0,07695
15/16N		0,02896		0,00084		0,01053		0,15177		0,23326
15/16K	0,6329	0,01833	1,5692	0,00132	0,2553	0,00269	0,2349	0,03565	0,2624	0,06121
03/15N		0,00814		0,00052		0,00770		0,14559		0,30355
03/15K	0,7792	0,00635	5,4264	0,00282	0,2813	0,00217	0,4090	0,05954	0,5946	0,18049

Patient ID	FC CDH1	2-ΔCT CDH1	FC EZH2	2-ΔCT EZH2	FC miR-101	2-ΔCT miR-101	FC miR-26b	2-ΔCT miR-26b	FC miR-200c	2-ΔCT miR-200c
20/17N		0,01075		0,00049		0,00452		0,15389		0,18557
20/17K	0,8351	0,00897	7,5162	0,00367	0,8293	0,00375	0,5548	0,08538	1,0425	0,19345
20/08N		1,08673		0,02856		0,00923		0,54337		0,73713
20/08K	0,8351	0,90752	1,9453	0,05555	1,0867	0,01003	0,1528	0,08304	0,1321	0,09740
12/16N		0,01807		0,00087		0,00534		0,12941		0,12763
12/16K	0,8827	0,01595	1,6702	0,00146	0,2073	0,00111	0,1768	0,02288	0,3345	0,04269
04/15N		0,02019		0,00016		0,00622		0,13397		0,17434
04/15K	0,9862	0,01992	13,5479	0,00217	0,1856	0,00115	0,1908	0,02556	0,3560	0,06207
12/09N		0,02816		0,00085		0,00491		0,30779		0,31208
12/09K	1,0140	0,02856	4,5002	0,00383	0,7474	0,00367	0,7684	0,23651	0,5035	0,15713
10/09N		0,13397		0,04039		0,02134		1,21419		1,00000
10/09K	1,2658	0,16958	0,0878	0,00354	0,3536	0,00755	0,5864	0,71203	0,7631	0,76313
08/15N		0,00760		0,00049		0,00333		0,03742		0,06426
08/15K	1,7411	0,01323	23,1029	0,01128	0,5586	0,00186	0,6690	0,02503	0,5510	0,03540
01/14N		0,01992		0,00044		0,01686		0,17678		0,22688
01/14K	1,7901	0,03565	2,6390	0,00116	0,1416	0,00239	0,1743	0,03082	0,5586	0,12674
13/16N		0,01060		0,00076		0,00260		0,04870		0,08362
13/16K	1,8277	0,01937	4,8232	0,00364	0,9202	0,00239	1,0497	0,05112	1,0210	0,08538
17/17N		0,00923		0,00032		0,01067		0,18816		0,17314
17/17K	1,8921	0,01746	0,5548	0,00018	0,4965	0,00530	0,8011	0,15073	0,3487	0,06037
08/07N		0,15713		0,00588		0,01833		0,31208		0,29525
08/07T	2,0849	0,32760	2,9079	0,01710	1,0000	0,01833	0,4965	0,15496	0,1830	0,05403
06/15N		0,01003		0,00345		0,00197		0,03125		0,10882
06/15K	2,3134	0,02320	0,2698	0,00093	2,4623	0,00484	3,5801	0,11188	1,4439	0,15713
11/16N		0,04239		0,01488		0,00458		0,08362		0,12500
11/16K	3,7321	0,15822	3,6808	0,05479	0,3950	0,00181	0,4444	0,03716	0,2483	0,03103
16/17N		0,01458		0,00042		0,01698		0,21169		0,27168
16/17K	5,6962	0,08304	21,1121	0,00879	0,4698	0,00798	0,2045	0,04328	1,1647	0,31644
14/16N		0,01698		0,00054		0,00861		0,20027		0,24486
14/16K	6,5887	0,11188	11,2356	0,00605	0,4033	0,00347	0,2535	0,05077	0,1358	0,03326
10/12N		0,05555		0,00125		0,00396		0,13870		0,07911
10/12K	14,0257	0,77916	13,5479	0,01698	1,6245	0,00643	2,1435	0,29730	4,5315	0,35849

References:

1. Chou, C.H.; Chang, N.W.; Shrestha, S.; Hsu, S.D.; Lin, Y.L.; Lee, W.H.; Yang, C.D.; Hong, H.C.; Wei, T.Y.; Tu, S.-J.; et al. miRTarBase 2016: updates to the experimentally validated miRNA-target interactions database. *Nucleic Acids Res.* **2016**, *44*, D239–D247.
2. Lewis, B.P.; Burge, C.B.; Bartel, D.P. Conserved Seed Pairing, Often Flanked by Adenosines, Indicates that Thousands of Human Genes are MicroRNA Targets. *Cell* **2005**, *120*, 15–20.
3. Friedman, R.C.; Farh, K.K.H.; Burge, C.B.; Bartel, D.P. Most mammalian mRNAs are conserved targets of microRNAs. *Genome Res.* **2008**, *19*, 92–105.
4. Agarwal, V.; Bell, G.W.; Nam, J.W.; Bartel, D.P. Predicting effective microRNA target sites in mammalian mRNAs. *Elife* **2015**, *4*.
5. Wong, N.; Wang, X. miRDB: an online resource for microRNA target prediction and functional annotations. *Nucleic Acids Res.* **2015**, *43*, D146–D152.
6. Wang, X. Improving microRNA target prediction by modeling with unambiguously identified microRNA-target pairs from CLIP-ligation studies. *Bioinformatics* **2016**, *32*, 1316–1322.
